# The Bacterial Defensin Resistance Protein MprF Consists of Separable Domains for Lipid Lysinylation and Antimicrobial Peptide Repulsion

**DOI:** 10.1371/journal.ppat.1000660

**Published:** 2009-11-13

**Authors:** Christoph M. Ernst, Petra Staubitz, Nagendra N. Mishra, Soo-Jin Yang, Gabriele Hornig, Hubert Kalbacher, Arnold S. Bayer, Dirk Kraus, Andreas Peschel

**Affiliations:** 1 Cellular and Molecular Microbiology Division, Interfaculty Institute of Microbiology and Infection Medicine, University of Tübingen, Tübingen, Germany; 2 Division of Infectious Diseases, Los Angeles Biomedical Research Institute at Harbor–University of California at Los Angeles (UCLA) Medical Center, Torrance, California, United States of America; 3 Medical and Natural Sciences Research Center, University of Tübingen, Tübingen, Germany; 4 David Geffen School of Medicine at UCLA, Los Angeles, California, United States of America; Dartmouth Medical School, United States of America

## Abstract

Many bacterial pathogens achieve resistance to defensin-like cationic antimicrobial peptides (CAMPs) by the multiple peptide resistance factor (MprF) protein. MprF plays a crucial role in *Staphylococcus aureus* virulence and it is involved in resistance to the CAMP-like antibiotic daptomycin. MprF is a large membrane protein that modifies the anionic phospholipid phosphatidylglycerol with l-lysine, thereby diminishing the bacterial affinity for CAMPs. Its widespread occurrence recommends MprF as a target for novel antimicrobials, although the mode of action of MprF has remained incompletely understood. We demonstrate that the hydrophilic C-terminal domain and six of the fourteen proposed *trans*-membrane segments of MprF are sufficient for full-level lysyl-phosphatidylglycerol (Lys-PG) production and that several conserved amino acid positions in MprF are indispensable for Lys-PG production. Notably, Lys-PG production did not lead to efficient CAMP resistance and most of the Lys-PG remained in the inner leaflet of the cytoplasmic membrane when the large N-terminal hydrophobic domain of MprF was absent, indicating a crucial role of this protein part. The N-terminal domain alone did not confer CAMP resistance or repulsion of the cationic test protein cytochrome *c*. However, when the N-terminal domain was coexpressed with the Lys-PG synthase domain either in one protein or as two separate proteins, full-level CAMP resistance was achieved. Moreover, only coexpression of the two domains led to efficient Lys-PG translocation to the outer leaflet of the membrane and to full-level cytochrome *c* repulsion, indicating that the N-terminal domain facilitates the flipping of Lys-PG. Thus, MprF represents a new class of lipid-biosynthetic enzymes with two separable functional domains that synthesize Lys-PG and facilitate Lys-PG translocation. Our study unravels crucial details on the molecular basis of an important bacterial immune evasion mechanism and it may help to employ MprF as a target for new anti-virulence drugs.

## Author Summary

Certain bacterial immune-evasion factors such as the MprF protein are highly conserved in many bacterial pathogens and represent attractive targets for new ‘anti-virulence’ drugs. MprF, initially discovered in the major human pathogen *Staphylococcus aureus*, protects bacteria against ‘innate human antibiotics’ such as the defensin peptides. In addition, MprF has recently been implicated in resistance to the new defensin-like antibiotic daptomycin. MprF modifies bacterial membrane lipids with the amino acid l-lysine, which leads to electrostatic repulsion of the membrane-damaging peptides. The molecular mechanism of MprF has remained largely unknown. We demonstrate that MprF represents a novel bifunctional type of enzyme. The N- and C-terminal domains of MprF are both required for mediating antimicrobial peptide resistance but they can be expressed as two separate proteins without loss of function indicating that they represent distinct functional modules. While the C-terminal domain accomplishes lipid lysinylation the N-terminal membrane-embedded domain is required to expose the lysine lipid at the outer surface of the bacterial membrane where it is able to repulse the antimicrobial peptides. These findings unravel the molecular basis of an important bacterial immune evasion mechanism and they may help to employ MprF as a target for new anti-virulence drugs.

## Introduction

In order to combat increasingly antibiotic-resistant bacteria such as *Staphylococcus aureus*, *Mycobacterium tuberculosis*, *Pseudomonas aeruginosa*, and enterococci new antimicrobial strategies based on compounds with anti-virulence or anti-fitness properties are increasingly in the focus of research efforts [1],[2]. Bacterial immune evasion mechanisms such as the *mprF* or *dltABCD*-encoded pathways are conserved over a wide range of bacterial species thereby representing attractive targets for broadly active antimicrobial compounds that would not kill the bacteria directly but render them susceptible to endogenous host defense molecules [3],[4].

The occurrence of closely related immune evasion factors in many bacterial pathogens is reflected by the conserved nature of the most critical antimicrobial host defense molecules. Defensins, cathelicidins, kinocidins, and related cationic antimicrobial peptides (CAMPs) are essential components of the antimicrobial warfare arsenals in humans, vertebrate and invertebrate animals, and even plants [5],[6]. Although peptide structures vary, overall structural features (cationic, amphipathic properties; often with γ-core motif) and modes of action (damage of microbial membrane-associated processes) are shared by most of these peptides [7]. CAMPs appear to take advantage of the fact that bacterial membranes are formed mostly by anionic phospholipids [4]. Conversely, the MprF and DltABCD proteins protect many bacterial pathogens against CAMPs by reducing the negative net charge of bacterial cell envelopes [3],[8]. The *dltABCD* operon products neutralize polyanionic teichoic acid polymers by esterification with d-alanine in many Gram-positive bacteria [9]. Detailed investigations on this pathway have recently led to the development of specific DltA inhibitors, which proved to be very effective anti-virulence drugs for eradication of bacterial infections [10],[11].

Much less is known on the MprF protein, which represents a particularly interesting antimicrobial drug target because of its presence in both, Gram-positive and Gram-negative bacteria [3]. MprF is a large integral membrane protein catalyzing the modification of the negatively charged lipid phosphatidylglycerol (PG) with l-lysine thereby neutralizing the membrane surface and providing CAMP resistance [12]–[14]. The resulting lysyl-phosphatidylglycerol (Lys-PG), described in pioneering biochemical studies in the 1960es [15],[16], is produced by an unusual pathway that uses PG and Lys-tRNA as substrate molecules [17]–[19]. The Lys-PG-biosynthetic enzyme has been identified only recently in *Staphylococcus aureus* and named multiple peptide resistance factor (*mprF*) because *mprF* mutants lacking Lys-PG are highly susceptible to CAMPs [12],[13]. The loss of Lys-PG in *mprF* mutants also led to CAMP susceptibility in *Listeria monocytogenes* [20], *Bacillus anthracis* [21], and *Rhizobium tropici* [22] thereby demonstrating a general role of MprF in bacterial immune evasion.

Recently, *mprF* point mutations or alterations in Lys-PG content became notorious for spontaneous resistance of *S. aureus* to daptomycin [23],[24]. This antibiotic has recently been approved as an antibiotic of last resort for the treatment of methicillin-resistant *S. aureus* (MRSA), which are responsible for a large proportion of hospital and, increasingly, community-acquired bacterial infections [25]. Daptomycin has a negative net charge but it is believed to have CAMP-like properties and mode of action upon binding of calcium ions [26]. In addition, MprF has been implicated in *S. aureus* susceptibility to the cationic antibiotics vancomycin, gentamycin, and moenomycin [27].

*mprF* expression is upregulated in staphylococci upon contact with CAMPs by the sensor/regulator system ApsRS [28],[29], which has also been named GraRS [30],[31]. Deletion of *mprF* has led to profoundly reduced virulence of several bacterial pathogens in animal models, which underscores the pivotal role of Lys-PG in bacterial fitness during colonization and infection [12],[20],[32],[33]. Accordingly, it is tempting to elucidate the molecular functions of MprF as a prerequisite for the development of small inhibitory molecules that would block Lys-PG biosynthesis and render a large number of bacterial pathogens highly susceptible to innate host defenses and cationic antibiotics such as daptomycin, glycopeptides, or aminoglycosides.

Here we demonstrate that MprF is a bifunctional protein composed of distinct and separable domains. While the C-terminal part of MprF is sufficient to synthesize Lys-PG the N-terminal hydrophobic protein domain is essential for efficient translocation of Lys-PG from the inner to the outer leaflet of the cytoplasmic membrane to reduce the bacterial affinity for CAMPs such as α-defensins, LL-37, daptomycin, or gallidermin.

## Results

### The first eight N-terminal *trans*-membrane segments (TMSs) of MprF are dispensable for Lys-PG synthase activity

**Figure 1:**
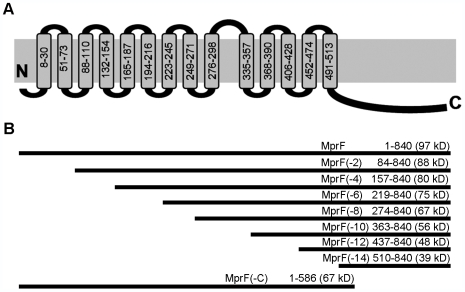
Structure of MprF and truncated proteins. A) Predicted *trans*-membrane topology of *S. aureus* MprF with amino acid positions predicted to form TMSs indicated. B) Truncated MprF proteins used to study the function of MprF. Length and calculated molecular weight of MprF variants are shown. Construction of plasmids is described in detail in Table S1.

Most MprF-like proteins are composed of large N-terminal hydrophobic domains followed by hydrophilic C-terminal domains [34] (Fig. S1). The hydrophilic portions exhibit much higher degrees of sequence similarity between different members of the MprF protein family [12] suggesting that this domain may play the most crucial role in Lys-PG biosynthesis. The hydrophobic domain of *S. aureus* MprF ranging from amino acid 1 to 509 is predicted to consist of 14 TMSs (Fig. 1A). In order to study whether the hydrophobic domain plays a role in Lys-PG biosynthesis the protein was shortened from the N-terminus in a step-wise manner by removing two TMSs at a time (Fig. 1B). The shortened proteins were expressed as N-terminal His-tag fusions and evaluated for their capacity to mediate Lys-PG production *in E. coli* BL21(DE3).

**Figure 2:**
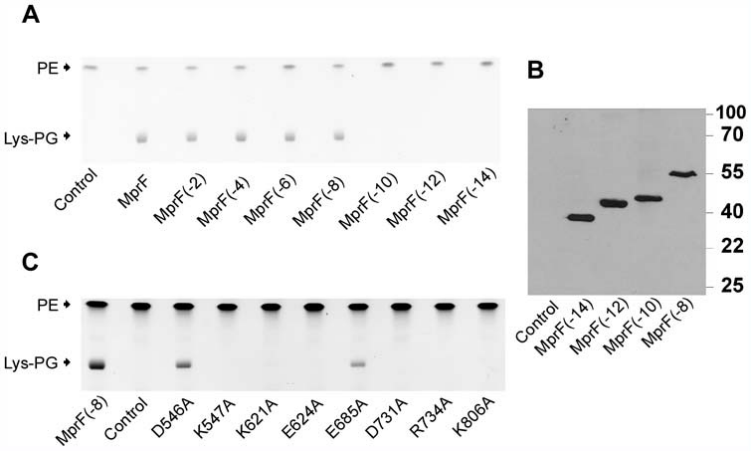
TLC and Western Blot analysis of *E. coli* expressing truncated or mutated variants of MprF. A) Polar lipids from strains containing expression plasmids without insert (control), with full-length *mprF*, or truncated *mprF* genes encoding proteins without the indicated TMS were separated by TLC and stained with the aminogroup-specific dye ninhydrin. B) The cytosolic fraction of *E. coli* strains expressing MprF(−14) and the membrane fractions of strains expressing MprF(−12), MprF(−10), MprF(−8) or containing the empty expression plasmid pET28 (control) were subjected to immunoblot analysis with a His-tag-specific antibody. The TMSs-containing proteins migrated slightly faster than expected, which is probably due to increased SDS binding capacity and/or incomplete unfolding of TMSs [49]. Molecular weight standard proteins are shown at the right margin. C) TLC analysis of *E. coli* strains expressing MprF variants with alanine exchanges. Polar lipids from strains containing the expression plasmid pET28a without insert (control), with unaltered *mprF*(−8), or with variants encoding proteins with the indicated amino acid exchanges were separated by TLC and stained with the aminogroup-specific dye ninhydrin. Positions of phosphatidylethanolamine (PE) and lysylphosphatidylglycerol (Lys-PG) are indicated in A) and C).

Deletion of the first eight TMSs of MprF from the N-terminus did not affect the ability of the protein to mediate Lys-PG production (Fig. 2A). However, further truncations abrogated Lys-PG production indicating that at least 6 TMSs are required for maintaining a functional enzyme and that the N-terminal domain of MprF may have a separate function. The presence and stability of the proteins was verified by Western-blotting with a His-tag-specific antibody. The shorter versions of MprF with no, two, four, or six predicted TMSs were detectable as singular similarly pronounced bands indicating that these proteins are largely stable (Fig. 2B). Longer versions of MprF including the full-length protein could not be visualized by Coomassie Blue staining or Western blotting even upon extensive variation of expression, isolation, and detection methods (data not shown), possibly because of inaccessibility of the N-terminal His-tag in these proteins. However, since all proteins ranging from MprF to MprF(−8) yielded similar levels of Lys-PG production the protein amounts and activities are unlikely to exhibit major differences.

Taken together, our data indicate that the N-terminal eight TMSs are dispensable for full-level Lys-PG synthesis while any further shortening completely abrogates the functionality of MprF.

### Lys-PG synthase activity depends on several conserved amino acid residues in the C-terminal part of MprF

Alignment of C-terminal MprF domains from different bacterial species revealed several conserved sequence motives, which may represent essential amino acids for substrate binding, enzymatic reaction, or folding into a stable protein of the Lys-PG synthase domain (Fig. S2). In order to evaluate the essential nature of such positions, eight highly conserved amino acid residues were exchanged with alanine residues by site-directed mutagenesis of the pET28mprF(−8) plasmid. Exchange of K547, K621, E624, D731, R734, and K806 led to complete abrogation of Lys-PG production (Fig. 2C). In contrast, replacement of E685 or D546 with alanine resulted in strongly or only slightly reduced Lys-PG production, respectively. The same results were obtained when the mutations were introduced into the full-length MprF protein (Fig. S3A). All the MprF(−8)-derived mutant proteins were detectable in Western Blots as singular protein bands that corresponded to the MprF(−8) protein (Fig. S3B) indicating that even the inactive proteins were stably produced in *E. coli*. Taken together, these data demonstrate essential roles of K547, K621, E624, D731, R734, and K806 for the enzymatic activity of MprF and less critical but important roles of D546 and E685.

### Expression of *mprF* (−8) in *S. aureus* Δ*mprF* leads to Lys-PG production but fails to confer resistance to cationic antimicrobial peptides

**Figure 3:**
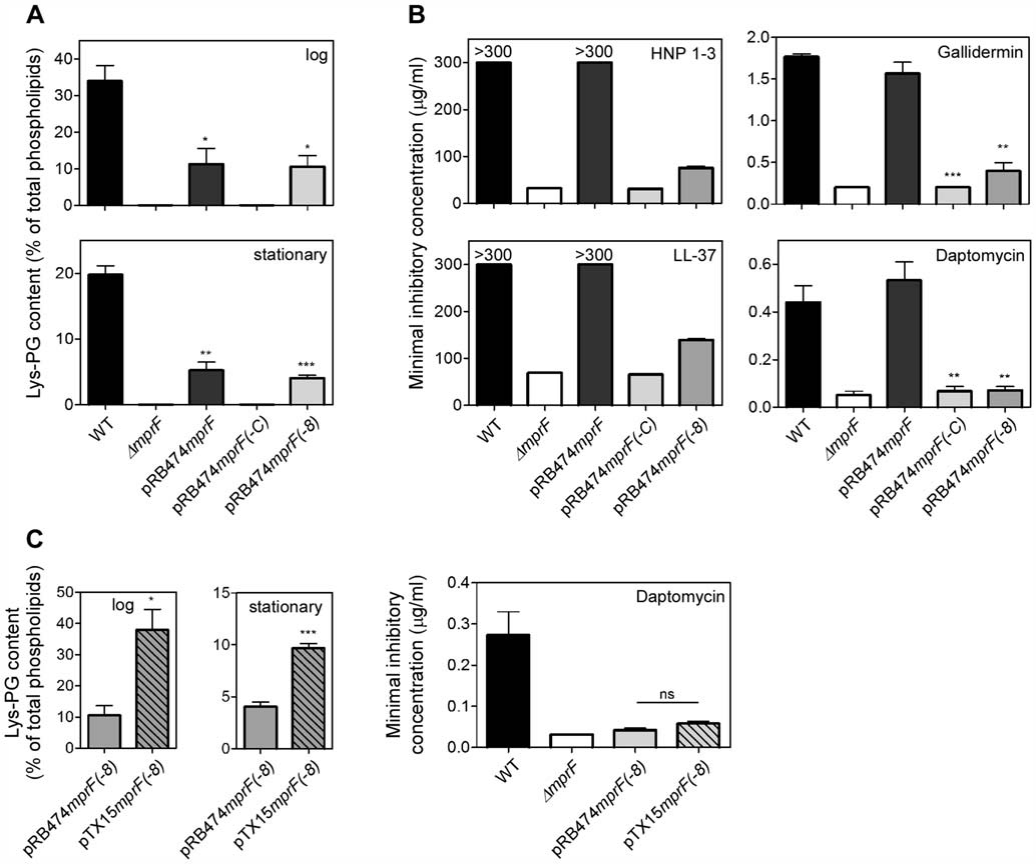
Impact on Lys-PG production and resistance to antimicrobial peptides of MprF variants in *S. aureus* Δ*mprF*. A) Lys-PG content in *S. aureus* wild-type (WT) or *ΔmprF* strains from logarithmic (log) or stationary growth phase containing the indicated plasmids were separated by TLC, stained with the phosphate groups-specific dye molybdenum blue, and quantified densitometrically. B) Minimal inhibitory concentrations (MICs) of CAMPs such as α-defensins HNP1-3, cathelicidin LL-37, gallidermin, and daptomycin. Means and SEM of three (HNP1-3, gallidermin, daptomycin) or two (LL-37) independent experiments are shown. MICs of HNP 1-3 and LL-37 for WT and *ΔmprF* with plasmid pRB474mprF were above the highest tested concentration of 300 µg/ml. Therefore, significances could only be calculated for gallidermin and daptomycin. C) Impact on Lys-PG content and daptomycin susceptibility of *ΔmprF* containing different expression vectors for the Lys-PG synthase domain MprF(−8). *, *P*<0.05; **, *P*<0.01; ***, *P*<0.001; ns, not significant versus WT (A), *ΔmprF* containing plasmid pRB474mprF (B), or *ΔmprF* containing plasmid pRB474mprF(−8) (C).

In order to investigate if MprF(−8) also mediates Lys-PG production in *S. aureus*, genes encoding the full-length and the MprF(−8) proteins were cloned in the *E. coli/Staphylococcus* shuttle expression vector pRB474 [35]. All the resulting plasmids led to Lys-PG production in *S. aureus* SA113 Δ*mpr*F (Fig. 3A) thereby reflecting the *E. coli* results. However, the Δ*mpr*F mutant with plasmid-encoded MprF or MprF(−8) did not reach the same level of Lys-PG as the wild-type strain. When the *S. aureus* strains were compared for susceptibility to CAMPs such as the α-defensins human neutrophil peptides 1–3 (HNP 1–3), the human cathelicidin LL-37, the bacteriocin gallidermin, or the antibiotic daptomycin, the *mpr*F mutant was much more susceptible than the wild-type strain (Fig. 3B), which is in agreement with previous findings [12],[23]. The strain containing the pRB474mprF(−8) plasmid was as susceptible to daptomycin as the *mpr*F deletion mutant or exhibited only slightly decreased susceptibilities as in the case of HNP1-3, LL-37, and gallidermin. However, only the full-length *mpr*F gene led to full resistance to the four peptides. This result indicates that the N-terminal hydrophobic domain of MprF is necessary for mediating efficient CAMP resistance despite the fact that it is dispensable for Lys-PG biosynthesis.

The presence of a basic level of Lys-PG seemed to be sufficient for full-level CAMP resistance provided that the N-terminal hydrophobic domain of MprF was not absent, while the total amounts of Lys-PG did not correlate with the levels of CAMP susceptibility (compare Lys-PG amounts and MIC values for WT and Δ*mpr*F containing plasmid pRBmprF). In order to verify this notion we cloned the minimal Lys-PG synthase domain MprF(−8) in the inducible staphylococcal expression vector pTX15, which has a higher copy number than pRB474 and permits xylose-inducible gene expression [35],[36]. *S. aureus* Δ*mpr*F with the resulting plasmid pTX15mprF(−8) had a 2.5–3.5-fold increased Lys-PG content as with the above described pRB474mprF(−8) (Fig. 3C). However, the two strains were inhibited by similarly low concentrations of daptomycin thereby confirming that Lys-PG production per se does not necessarily cause CAMP resistance, irrespective of the produced amounts of Lys-PG.

### Both, the hydrophobic domain of MprF and the Lys-PG synthase domain are required for CAMP resistance but they do not need to be covalently linked

**Figure 4:**
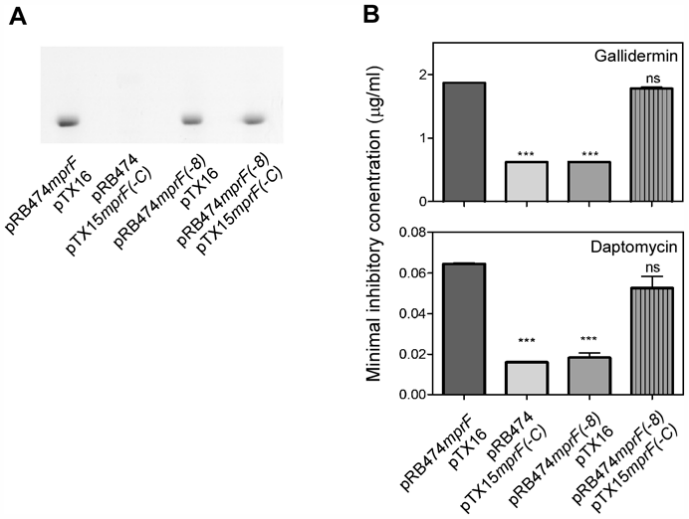
Impact on Lys-PG production and resistance to antimicrobial peptides of MprF(−8) and MprF(−C) expressed *in trans*. The two protein domains were expressed on separate plasmids [pRB474mprF(−8) and pTX15mprF(−C)] in *S. aureus ΔmprF*. A) Polar lipids were separated by TLC and stained with the aminogroup-specific dye ninhydrin. B) Minimal inhibitory concentrations of gallidermin and daptomycin. pRB474 and pTX16 are empty control plasmids. Means and SEM of three independent experiments are shown. ***, *P*<0.001; ns, not significantly different versus *S. aureus ΔmprF* containing plasmid pRB474mprF and pTX16.

In order to explore the role of the N-terminal domain of MprF in CAMP resistance the *mprF*(−C) gene encoding only the 14 TMSs without the hydrophilic C-terminal domain was expressed in *S. aureus* Δ*mpr*F. Of note, the resulting strain did not show resistance to any of the tested CAMPs compared to the Δ*mpr*F mutant (Fig. 3B) indicating that this protein domain alone cannot protect the bacteria from CAMPs and depends on the Lys-PG synthase. In order to evaluate if the two domains need to be fused or can be separated to achieve CAMP resistance, the *mprF*(−C) gene was cloned in pTX15, which is compatible with pRB474-derived plasmids. The resulting plasmid pTX15mprF(−C) or the empty control plasmid pTX16 were introduced into *S. aureus* Δ*mpr*F bearing pRB474mprF(−8). The MIC values of daptomycin reached much lower levels in the presence of two plasmids compared to the experiments described above, which is probably due to increased stress imparted on the two plasmids-containing bacteria. Notably, when MprF(−C) was co-expressed with MprF(−8) *in trans* it conferred full CAMP resistance, which reached the same level as the unchanged MprF protein (Fig. 4B). Thus, the hydrophobic domain of MprF can only mediate CAMP resistance if the synthase domain is present but the two proteins can be separated and do not need to be covalently linked.

### The N-terminal hydrophobic domain of MprF is required for efficient translocation of Lys-PG to the outer leaflet of the cytoplasmic membrane

**Figure 5:**
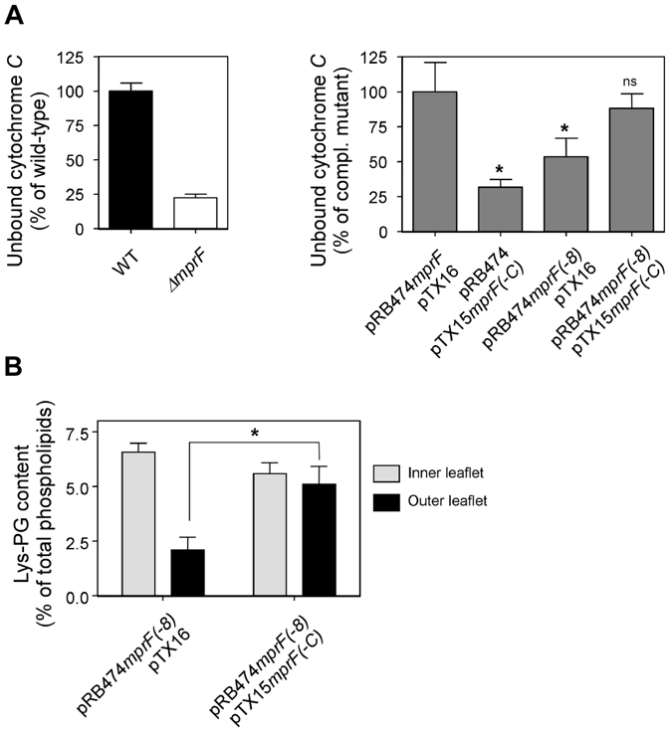
Impact of the hydrophobic N-terminal domain of MprF on the ability of Lys-PG to repulse cationic cytochrome *c* and to reach the outer leaflet of the cytoplasmic membrane. A) The capacities of *S. aureus* wild-type (WT) and *ΔmprF* (left panel) or *ΔmprF* containing the indicated plasmids (right panel) to bind cytochrome *c* were compared. B) Inner and outer-leaflet localization of Lys-PG in *ΔmprF* bearing the indicated plasmids was determined by analyzing the ability of the membrane-impermeable fluorescent dye fluorescamine to react with Lys-PG. pRB474 and pTX16 are empty control plasmids. Means and SEM of three (A) and four to eight replicas from two (B) independent experiments are shown. *, *P*<0.05; **, *P*<0.01; ns, not significantly different versus *S. aureus* WT (A, left panel) or *ΔmprF* containing plasmids pRB474mprF and pTX16 (A, right panel).

While Lys-PG is synthesized at the inner leaflet of the cytoplasmic membrane where the Lys-tRNA donor substrate is available, the lipid can only exert its role in CAMP resistance when present at the outer leaflet of the membrane, where the antimicrobial peptides are encountered. In order to evaluate the possibility that the N-terminal hydrophobic domain of MprF facilitates the translocation and exposure of Lys-PG at the outer leaflet of the membrane, we first investigated the impact of MprF(−C) on surface charge neutralization and concomitant repulsion of cationic peptides [12]. A previously described assay based on the bacterial binding capacity of the small red-coloured cationic protein cytochrome *c* was used for this approach [37]. As expected, the *mprF* mutant had a profoundly higher capacity to bind cytochrome *c* as the wild-type strain, which reflects the highly negatively charged membrane surface in the absence of Lys-PG (Fig. 5A). Likewise, expression of MprF(−C) or of the synthase domain MprF(−8) in *S. aureus* Δ*mpr*F led to substantially reduced repulsion of cytochrome *c* compared to the unaltered MprF. However, when the two protein parts were simultaneously expressed *in trans* they led to the same level of cytochrome *c* repulsion as expression of the unaltered MprF protein (Fig. 5A). These results parallel the inability of MprF(−8) and MprF(−C) to confer CAMP resistance individually and they confirm that the two proteins have complementary functions that can be physically separated.

The ability of the N-terminal hydrophobic domain of MprF to facilitate the translocation of Lys-PG from the inner to the outer leaflet of the cytoplasmic membrane was verified by comparing the capacity of Lys-PG to be modified by the aminogroups-reactive, membrane-impermeable fluorescent dye fluorescamine in the absence or presence of MprF(−C). This assay has been developed to analyze the distribution of amino-phospholipids between inner or outer leaflets of membranes [38],[39] and has been successfully used to compare Lys-PG distribution in spontaneously CAMP-resistant *S. aureus* mutants [24],[40]. When only the synthase domain of MprF was expressed in *S. aureus* Δ*mpr*F, only a small fraction of total Lys-PG was found in the outer leaflet (Fig. 5B). However, when MprF(−C) was coexpressed with the synthase domain, the amount of Lys-PG in the outer leaflet was strongly increased and reached a similar level as in the inner leaflet. Thus, the N-terminal hydrophobic domain of MprF is required for efficient translocation of Lys-PG.

## Discussion

While the anionic phospholipids PG and cardiolipin are produced by virtually any bacterial species, zwitterionic or cationic lipids such as PE or Lys-PG, respectively, are produced only by certain groups of bacteria [41]. Despite extensive research efforts the actual roles of the various phospholipids, their biosynthesis, turnover, and regulation, have remained incompletely understood. Of note, the same holds true for the identity, specificity, and mode of action of proposed bacterial translocator proteins required to flip the lipids, which are generated at the inner cytoplasmic membrane leaflet, to the outer leaflet. MprF represents the paradigm of a new class of bifunctional lipid-biosynthetic enzymes mediating the transfer of amino acids to anionic phospholipids. While the *S. aureus* MprF mediates exclusively the biosynthesis of Lys-PG, the MprF homolog from *L. monocytogenes* seems to confer both, Lys-PG and Lys-cardiolipin biosynthesis [20]. MprF homologs from *C. perfringens* and *P. aeruginosa* have been shown to mediate Ala-PG production [34],[42]. Our study represents a basis for investigating the determinants of substrate specificity of MprF.

Six of the 14 TMSs plus the hydrophilic C-terminal domain were sufficient to mediate Lys-PG production in *E. coli* or *S. aureus*. The levels of Lys-PG production varied between *S. aureus* strains with different plasmid vectors and promoters used to express MprF or MprF variants but the level of Lys-PG did not correlate with the level of CAMP resistance indicating that only a basic amount of Lys-PG is sufficient for repulsing antimicrobial peptides provided that the lipid is translocated to the outer leaflet of the membrane. It is amazing that the Lys-PG synthase whose active center is probably located in the hydrophilic domain of MprF with its many conserved amino acid positions requires so many TMSs to function since one or two such segments should be enough to anchor the hydrophilic C-terminus in the membrane. One might speculate that six TMSs are required to embrace a PG substrate molecule and fit it into a position that may allow its lysinylation. It should be noted that even the MprF homolog with the shortest integral membrane domain found in *Mycobacterium tuberculosis* is predicted to harbor six TMSs (data not shown), which suggests that the dependence on six TMSs is a general property of MprF-like enzymes.

Previous studies on *in vitro* Lys-PG biosynthesis with artificially altered aminoacyl tRNAs have demonstrated that the Lys-PG synthase recognizes features of both, the tRNA and of the bound amino acid [17],[19]. Accordingly, the lysyl group could not be transferred to PG when it was attached to a cysteinyl tRNA. However, it did not matter whether the tRNA came from *S. aureus* or from another bacterial species such as *E. coli* [18]. We identified six conserved amino acids in the C-terminal domain of MprF as essential for Lys-PG biosynthesis while exchange of two other amino acid positions led to reduced Lys-PG production. All these positions are also conserved in MprF homologs with Ala-PG synthase activity (Fig. S2), which suggests that they are not involved in specific recognition of the aminoacyl tRNA precursor and may rather play crucial roles in the enzymatic process or in non-specific binding of the substrate. Irrespective of the tRNA structure the substrate-binding domain of MprF may need basic properties to interact with the polyanionic ribonucleic acid. Accordingly, four of the six identified essential amino acid position represent cationic arginine or lysine residues that may participate in binding of tRNA phosphate groups.

**Figure 6:**
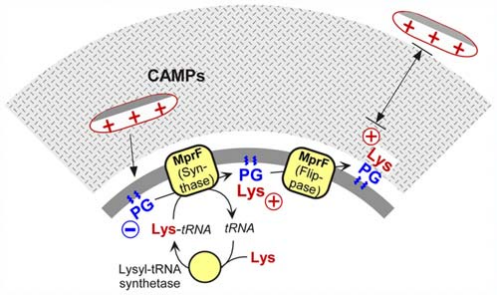
Model for the mode of MprF-mediated bacterial CAMP resistance. Lys-PG is synthesized from Lys-tRNA and PG by the synthase domain of MprF. Lys-PG can only neutralize the outer surface of the membrane upon translocation to the outer cytoplasmic membrane leaflet, which is facilitated by the large N-terminal integral membrane domain of MprF.

A most intriguing finding of our study was the fact that Lys-PG production on its own did not lead to CAMP resistance but depended on the large N-terminal integral membrane domain of MprF. Lys-PG mediates CAMP resistance by repulsing the cationic peptides from the outer surface of the membrane, which is only possible upon translocation of the lipid to the outer leaflet (Fig. 6). Of note, Lys-PG could only alter the membrane surface charge considerably in the presence of the N-terminal integral membrane domain indicating that this part of MprF is required for this lipid to reach the outer leaflet of the membrane. Moreover, Lys-PG could only be labeled efficiently by the membrane-impermeable dye fluorescamine in the presence of the N-terminal hydrophobic domain of MprF, which confirms the critical role of this protein part in Lys-PG translocation. Thus, MprF does not only synthesize Lys-PG but also accomplishes translocation of Lys-PG from the inner to the outer surface of the membrane. These two functions are allocated in the C-terminal and N-terminal domains of MprF, respectively, and can be separated into two functional proteins (Fig. 6).

While lipid translocators have been investigated to some extent in eukaryotic cells [43], such proteins have been proposed but hardly described in bacteria. It is possible that the bacterial house-keeping translocator(s) are more specific for the standard anionic phospholipids PG and cardiolipin, while a cationic lipid such as Lys-PG may require a dedicated translocator. It remains unclear why a small fraction of Lys-PG was detectable in the outer leaflet of the cytoplasmic membrane even in the absence of the flippase domain of MprF. Phospholipids may be able to flip spontaneously with low efficiency as proposed recently [44] or one of the house-keeping flippases may have residual activity for Lys-PG. Lipid translocators have been classified into energy-dependent (flippases or floppases) and energy-independent (scramblases) transporters [43]. MprF does not contain conserved ATP-binding or other sequence motives indicative of energy consumption. Therefore, it remains unclear if MprF can accomplish an asymmetric distribution of Lys-PG. Nevertheless, recent studies suggest that Lys-PG can be asymmetrically distributed between the inner and outer leaflets of the membrane in *S. aureus* depending on the individual strain background [24].

The increasing resistance of major bacterial pathogens raises the specter of untreatable infections as in the pre-antibiotics era. MRSA are now more and more prevalent in the community and only a few antibiotics of last resort such as daptomycin have remained effective against such highly pathogenic *S. aureus* clones. As *S. aureus* can overcome even daptomycin by simple point mutations in *mprF* new strategies for antibacterial chemotherapy are urgently needed. Inhibitors for highly conserved immune evasion factors such as *mprF* that would render a wide range of bacteria susceptible to endogenous human defense mechanisms and cationic antibiotics such as daptomycin should be increasingly considered. Our study represents a basis for more detailed investigations on the structure and mode of action of MprF-like aminoacylphospholipid synthases and they should enable the systematic search for inhibitors for this class of enzymes.

## Materials and Methods

The plasmids and strains constructed in this study are listed in Table S1 and primers are listed in Table S2. Construction of plasmids, growth conditions, alignment and prediction of MprF structure, Western-blot analysis, lipid extraction, and analysis of Lys-PG distribution are described in Text S1.

For detection of Lys-PG appropriate amounts of polar lipid extracts were spotted onto silica 60 F254 HPTLC plates (Merck, Darmstadt, Germany) using a Linomat 5 sample application unit (Camag, Berlin, Germany) and developed with chloroform/methanol/water (65∶25∶4, by vol.) in an automatic developing chamber ADC 2 (Camag, Berlin, Germany). Amino groups or phosphate groups-containing lipids were selectively stained with ninhydrin spray (0.3 g ninhydrin dissolved in 100 ml 1-butanol and 3 ml 100% acetic acid) or molybdenum blue spray (Sigma). Integrated lipid spot intensities of molybdenum blue-stained phospholipids were determined by ImageJ (http://rsbweb.nih.gov/ij/). MIC values of gallidermin, HNP1-3, and LL-37 were determined by diluting bacteria from overnight cultures to an OD_600 nm_ of 0.05 −0.1 in fresh MHB medium (gallidermin) or half-concentrated MHB (HNP1-3 and LL-37) containing serial dilutions of antimicrobial peptides as described recently [45]. Gallidermin was kindly provided by Friedrich Götz. HNP1-3 was isolated from human neutrophils and purified by reversed-phase high-performance liquid chromatography (RP-HPLC) as described previously [37]. LL-37 was synthesized by solid-phase peptide synthesis and purified by RP-HPLC [46]. Susceptibility to daptomycin was determined by epsilometer test (E test) in the presence of CaCl_2_ according to the manufacturer's advise (AB Biodisk) [47]. Differences in bacterial capacity to repulse cationic proteins were determined by comparing binding of the red-coloured cationic protein cytochrome *c* as described recently [37],[48].

### List of SwissProt accession numbers

Q2G2M2: *Staphylococcus aureus* MprF; Q5HPI1: *Staphylococcus epidermidis* MprF homolog; C0H3X7: *Bacillus subtilis* MprF homolog; C0X347: *Enterococcus faecalis* MprF homolog; Q8DWT2: *Streptococcus agalactiae* MprF homolog; Q71YX2: *Listeria monocytogenes* MprF homolog; Q88YQ7: *Lactobacillus plantarum* MprF homolog; Q8FW76: *Brucella suis* MprF homolog; Q9I537: *Pseudomonas aeruginosa* MprF homolog; Q0SSM7 and Q0STHJ7: *Clostridium perfringens* MprF homologs.

## Supporting Information

Text S1 Supplementary Materials and Methods.
(0.07 MB PDF)

Figure S1 Kyte-Doolittle hydrophobicity profile of *S. aureus* MprF.
(2.06 MB TIF)

Figure S2 Alignment of the hydrophilic C-terminal parts of MprF proteins of various bacterial species. Partially and completely conserved positions are boxed in gray and black, respectively. Amino acid positions exchanged by site-directed mutagenesis are indicated. The following proteins were compared (SwissProt accession numbers are given in brackets): Sa: *S. aureus* (Q2G2M2), Se: *S. epidermidis* (Q5HPI1), Bs: *Bacillus subtilis* (C0H3X7), Ef: *Enterococcus faecalis* (C0X347), Sag: *Streptococcus agalactiae* (Q8DWT2), Lm: *Listeria monocytogenes* (Q71YX2), Lp: *Lactobacillus plantarum* (Q88YQ7), Brs: *Brucella suis* (Q8FW76), PS: *Pseudomonas aeruginosa* (Q9I537), Cp1 and Cp2: *Clostridium perfringens* (Q0SSM7 and Q0STHJ7, respectively). The *C. perfringes* Cp1 protein mediates Lys-PG biosynthesis while the *C. perfringes* Cp2 and the *P. aeruginosa* protein mediate Ala-PG biosynthesis [15],[16].
(9.54 MB TIF)

Figure S3 Western Blot analysis of *E. coli* with pET28*mprF*(−8) derivatives containing the indicated amino acid exchanges and TLC analysis of *E. coli* with mutated full-length *mprF* genes cloned in pBAD containing the indicated amino acid exchanges. (A) Proteins from crude lysates were subjected to immunoblot analysis with a His-tag-specific antibody. Molecular weight standard proteins are shown at the right margin. (B) Polar lipids from the indicated strains were separated by TLC and stained with the aminogroup-specific dye ninhydrin. Positions of PE and Lys-PG are indicated.
(3.21 MB TIF)

Table S1 Plasmids for expression of truncated or mutated MprF variants.
(0.02 MB PDF)

Table S2 Primers used for plasmid construction.
(0.02 MB PDF)
