# Supplementary material for: The Bacterial Defensin Resistance Protein MprF Consists of Separable Domains for Lipid Lysinylation and Antimicrobial Peptide Repulsion
Source: PLoS Pathog. 2009 Nov 13;5(11):e1000660. doi: 10.1371/journal.ppat.1000660 (PMC2774229; doi:10.1371/journal.ppat.1000660)
Supplement: Text S1 — Supplementary Materials and Methods. (0.07 MB PDF) [file ppat.1000660.s001.pdf]

(Revised manuscript)

Supporting Information (Ernst et al.)

SI Text

Supplementary Materials and Methods.

Bacterial strains, and growth conditions. *E. coli* DH5α, *E. coli* BL21 DE3, and *S. aureus* SA113 (ATCC 35556) are frequently used laboratory strains. The Lys-PG-deficient *S. aureus* SA113 Δ*mprF* mutant has been described recently [1]. *E. coli* or *S. aureus* strains were grown in LB broth (1% tryptone, 0.5% yeast extract, 0.5% NaCl) or BM broth (1% tryptone, 0.5% yeast extract, 0.5% NaCl, 0.1% K_2HPO_4, 0.1% glucose), respectively, supplemented with appropriate antibiotics unless otherwise noted.

Prediction of MprF structure and similarity. The *trans*-membrane topology of MprF was predicted with the SOSUI program (engine ver.1.11; http://bp.nuap.nagoya-u.ac.jp/sosui/). MprF was compared with homologous protein sequences using the DNASIS MAX 2.05 software (Hitachi Software Engineering) to define highly conserved amino acid residues in the hydrophilic C-terminal domain of MprF. Kyte and Doolittle hydrophobicity profiles of MprF were generated by Protscale, a protein identification and analysis tool on the ExPASy server (http://www.expasy.ch/tools/protscale.html).

Expression and mutagenesis of mprF in E. coli. *S. aureus mpr*F or *mpr*F truncations were PCR-amplified using the primers described in Table S2. Primers were modified to introduce restriction sites as listed in Table S1 to allow ligation of the PCR products to corresponding restriction sites of the polylinker in pET28a [2] resulting in *in-frame* fusion of *mpr*F sequences with the plasmid-encoded ribosomal binding site plus N-terminal His-tag. The original *mpr*F stop codon was maintained in cloned PCR products. For *mpr*F expression in pBAD [3] primers contained restriction sites to allow *in-frame* ligation into the plasmid's polylinker resulting in N-terminal fusion with the plasmid-encoded ribosomal binding site. All PCR-amplified DNA

fragments were verified by sequencing. *E. coli* and *S. aureus*-specific molecular techniques followed established protocols [4–6]. Selected amino acids of MprF were replaced with alanine by site-directed mutagenesis using the Quickchange kit (Stratagene, La Jolla, CA, USA). Primers used for mutagenesis are listed in Table S2. Plasmids pBADmprF and pET28mprF(-8) were used as templates for the mutagenesis and amplification procedures. The resulting plasmids and proteins are given in Table S1.

Cloning of mprF in the Staphylococcus-specific plasmids pRB474 or pTX15. *mprF* and truncated genes were cloned in the *E. coli* / *staphylococcus*-specific shuttle plasmid pRB474 [7] in such a way that the 5' end was downstream of the plasmid-encoded constitutive *veg* promoter as described in Table S1. In parallel, *mprF*(-C) and *mprF*(-8) were cloned in the *staphylococcus*-specific expression vector pTX15 [8] in the right orientation to permit expression from the xylose-inducible plasmid-encoded *xyl* promoter (strains were induced with 0.5% xylose). Spontaneous homologous recombination of pRB474 and pTX15-derived plasmids containing overlapping portions of *mprF* was excluded by restriction fragment length analysis and PCR. Empty plasmids pTX16 [8] or pRB474 were used in certain control strains.

Isolation of polar lipids. LB was inoculated with overnight cultures of *E. coli* strains at a final OD_{600} of 0.1 and incubated at 37°C for 1 h. To allow for expression of *mpr*F and derivatives, 0.2% arabinose (and antibiotic ampicillin) in case of pBAD derivatives or 0.5 mM IPTG (and antibiotic kanamycin) in case of pET28 derivatives, respectively, was added and cultures were incubated for further 2 h. Lipids were isolated from *S. aureus* cultures grown to the logarithmic phase (3 h) or stationary phase (18 – 20 h) in BM medium or in case of the strains harbouring the pTX15-derived plasmid in Mueller Hinton Broth containing 0.5% xylose. Polar lipids were extracted with chloroform/methanol (2:1, by vol.) by the Bligh-Dyer procedure [9], vacuum-dried, and dissolved in chloroform/methanol (2:1, by vol.).

Western-blot detection of MprF and MprF-derived proteins. To prepare cytoplasmic and membrane proteins for Western blot analysis, *E. coli* strains were grown and gene expression

was induced as described above. Proteins from cytoplamic and membrane fractions were isolated using a modified protocol of the ProteoExtract Transmembrane Protein Extraction Kit (Calbiochem). Briefly, cells were washed with PBS and disrupted with extraction buffer I from the kit supplemented with lysozyme and DNase (each 200 U/ml). Membrane proteins were separated from soluble proteins by centrifugation. The membrane fraction was treated with the supplied *trans*-membrane protein solubilization agent TM-PEK Reagent A and after incubation for two hours and another centrifugation step, the solubilized integral membrane protein fraction in the supernatant was subjected to Western blot analysis. Proteins from the various fractions were separated by SDS-PAGE on Tris-glycine gels with 10% acrylamide and stained with Coomassie Blue or subjected to Western blot analysis using a monoclonal anti-His-tag antibody (Penta-His Antibody, Qiagen) and a HRP-coupled goat anti-mouse secondary antibody (Merck). Polyacrylamide gel electrophoresis and Western blot analysis were carried out according to standard procedures [10]. Other antibodies that were tested to detect larger variants of MprF but failed to yield signals included the Tetra-His antibody (Invitrogen), Anti-His6 Antibody (Roche), Anti-His mAB antibody (Genscript), and the InVision His-tag In-gel stain (Invitrogen). In addition, various substances were tested in different concentrations and combinations to try solubilize MprF and larger variants includng triton X 100, methyl-β-cyclodextrin, sarcosyl, n-dodecyl-β-D-maltosid, urea, guanidiumthiocyanate, guanidiumhydrochloride and CHAPS.

Lys-PG distribution. Fluorescamine, a fluorescent probe, which specifically labels surface-exposed (outer leaflet), positively-charged amino-phospho lipids, was used to assay for Lys-PG distribution in the inner or outer leaflets of the cytoplasmic membrane [11–14]. Bacteria were grown to stationary phase and harvested as described previously [14]. Cells (0.6 g wet weight) were suspended in 3 ml of buffer B (100 mM potassium phosphate, 600 mM potassium chloride, pH 8.2) (~1 x 10^8 cells/ml), transferred to a 25-ml glass conical flask and cooled to 4°C with gentle swirling. To the cell pellet, 90 µl fluorescamine solution (0.52 M) in dehydrated dimethylsulfoxide was added drop-wise, with constant swirling over 30 s. The reaction was

stopped by the addition of 3 ml of 1 M ammonia in 600 mM potassium chloride. The suspension was centrifuged (3000 x *g*); the pellet was washed four times at 4°C with buffer C (200 mM potassium acetate, 600 mM potassium chloride, pH 4.5) until the supernatant was free of color, and then subjected to two-dimensional thin-layer chromatography as described previously [14]. Fluorescamine labeling of outer-leaflet Lys-PG was detected by using a UV detector (365 nm excitation). Of note, once bound to Lys-PG, fluorescamine alters its mobility characteristics and attenuates its ability to be detected by ninhydrin staining [14]. After detection of the fluorescamine-labeled Lys-PG, its relative content (normalized with respect to total phospholipids) was quantified by measuring the phosphate content by a colorometric assay [9].

Statistical methods. Statistical analyses were performed with the Prism 4.0 package (GraphPad Software) and the between-groups differences were analyzed for significance with the two-tailed Student's *t*-test. A *P* value $\leq$ 0.05 was considered statistically significant.

SI Reference List

1. Peschel A, Jack RW, Otto M, Collins LV, Staubitz P, et al. (2001) *Staphylococcus aureus* resistance to human defensins and evasion of neutrophil killing via the novel virulence factor MprF is based on modification of membrane lipids with L-lysine. J Exp Med 193: 1067-1076.

2. Rosenberg AH, Lade BN, Chui DS, Lin SW, Dunn JJ, et al. (1987) Vectors for selective expression of cloned DNAs by T7 RNA polymerase. Gene 56: 125-135.

3. Tormay P, Coates AR, Henderson B (2005) The intercellular signaling activity of the *Mycobacterium tuberculosis* chaperonin 60.1 protein resides in the equatorial domain. J Biol Chem 280: 14272-14277.

4. Sambrook J, Fritsch EF, Maniatis T (1989) Molecular cloning: a laboratory manual. Cold Spring Harbor, New York: Cold Spring Harbor Laboratory Press.

5. Foster TJ (1998) Molecular genetic analysis of staphylococcal virulence. Methods Microbiol 27: 433-454.

6. Novick RP (1991) Genetic systems in staphylococci. Methods Enzymol 204: 587-636.

7. Brückner R (1992) A series of shuttle vectors for *Bacillus subtilis* and *Escherichia coli*. Gene 122: 187-192.

8. Peschel A, Ottenwälder B, Götz F (1996) Inducible production and cellular location of the epidermin biosynthetic enzyme EpiB using an improved staphylococcal expression system. FEMS Microbiol Lett 137: 279-284.

9. Bligh EG, Dyer WJ (1959) A rapid method of total lipid extraction and purification. Can J Biochem Physiol 37: 911-917.

10. Ausubel FM, Brent R, Kingston RE, Moore DD, Seidman JG et al. (1990) Current protocols in molecular biology. New York, NY: John Wiley and Sons, Inc.

11. Balasubramanian K, Gupta CM (1996) Transbilayer phosphatidylethanolamine movements in the yeast plasma membrane. Evidence for a protein-mediated, energy-dependent mechanism. Eur J Biochem 240: 798-806.

12. Dogra S, Krishnamurthy S, Gupta V, Dixit BL, Gupta CM, et al. (1999) Asymmetric distribution of phosphatidylethanolamine in C. albicans: possible mediation by CDR1, a multidrug transporter belonging to ATP binding cassette (ABC) superfamily. Yeast 15: 111-121.

13. Jones T, Yeaman MR, Sakoulas G, Yang SJ, Proctor RA, et al. (2008) Failures in clinical treatment of Staphylococcus aureus Infection with daptomycin are associated with alterations in surface charge, membrane phospholipid asymmetry, and drug binding. Antimicrob Agents Chemother 52: 269-278.

14. Mukhopadhyay K, Whitmire W, Xiong YQ, Molden J, Jones T, et al. (2007) In vitro susceptibility of Staphylococcus aureus to thrombin-induced platelet microbicidal protein-1 (tPMP-1) is influenced by cell membrane phospholipid composition and asymmetry. Microbiology 153: 1187-1197.
