# Supplementary figures and images for: The Bacterial Defensin Resistance Protein MprF Consists of Separable Domains for Lipid Lysinylation and Antimicrobial Peptide Repulsion
Source: PLoS Pathog. 2009 Nov 13;5(11):e1000660. doi: 10.1371/journal.ppat.1000660 (PMC2774229; doi:10.1371/journal.ppat.1000660)

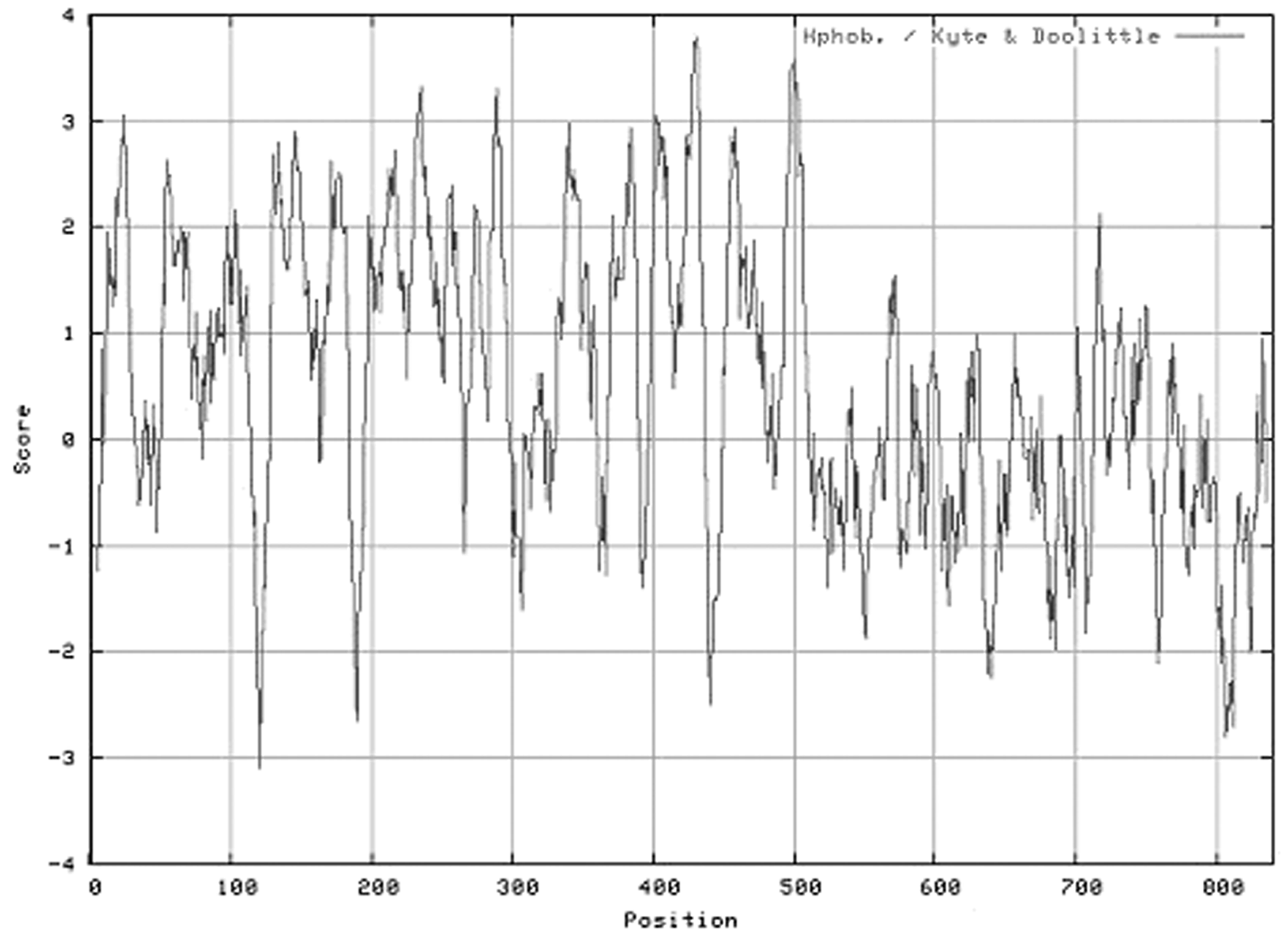

Supplement: Figure S1 — Kyte-Doolittle hydrophobicity profile of S. aureus MprF. (2.06 MB TIF) [file ppat.1000660.s002.tif]

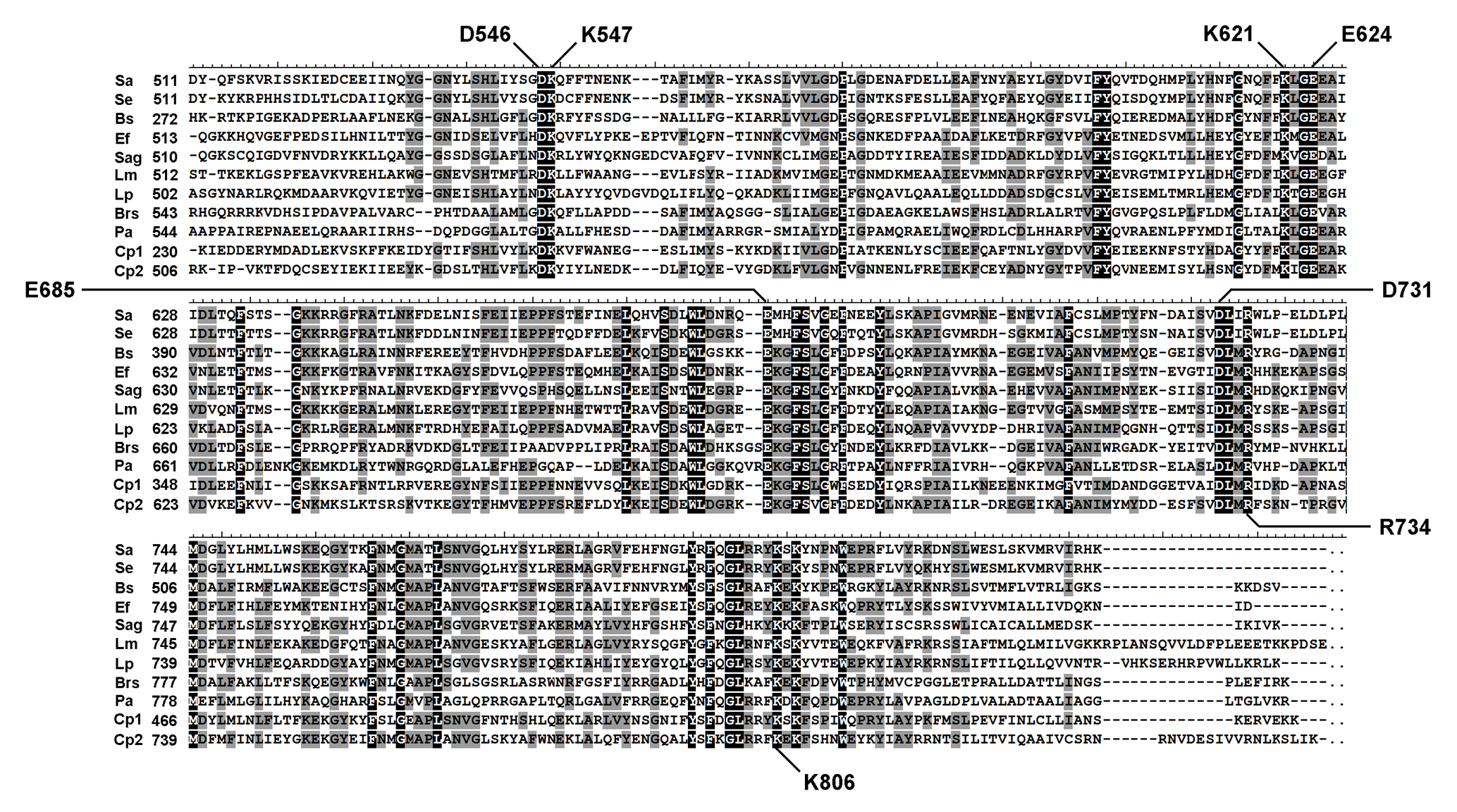

Supplement: Figure S2 — Alignment of the hydrophilic C-terminal parts of MprF proteins of various bacterial species. Partially and completely conserved positions are boxed in gray and black, respectively. Amino acid positions exchanged by site-directed mutagenesis are indicated. The following proteins were compared (SwissProt accession numbers are given in brackets): Sa: S. aureus (Q2G2M2), Se: S. epidermidis (Q5HPI1), Bs: Bacillus subtilis (C0H3X7), Ef: Enterococcus faecalis (C0X347), Sag: Streptococcus agalactiae (Q8DWT2), Lm: Listeria monocytogenes (Q71YX2), Lp: Lactobacillus plantarum (Q88YQ7), Brs: Brucella suis (Q8FW76), PS: Pseudomonas aeruginosa (Q9I537), Cp1 and Cp2: Clostridium perfringens (Q0SSM7 and Q0STHJ7, respectively). The C. perfringes Cp1 protein mediates Lys-PG biosynthesis while the C. perfringes Cp2 and the P. aeruginosa protein mediate Ala-PG biosynthesis [15],[16]. (9.54 MB TIF) [file ppat.1000660.s003.tif]

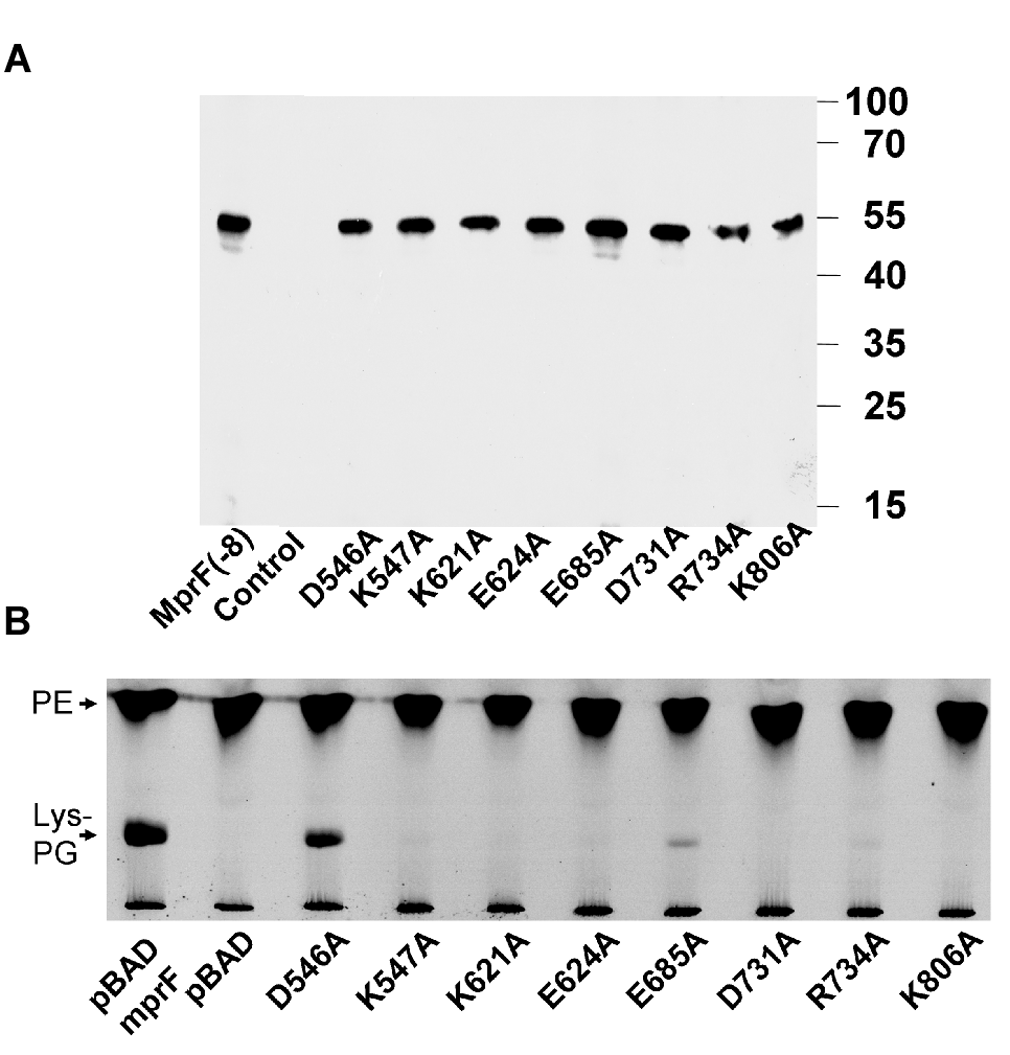

Supplement: Figure S3 — Western Blot analysis of E. coli with pET28mprF(−8) derivatives containing the indicated amino acid exchanges and TLC analysis of E. coli with mutated full-length mprF genes cloned in pBAD containing the indicated amino acid exchanges. (A) Proteins from crude lysates were subjected to immunoblot analysis with a His-tag-specific antibody. Molecular weight standard proteins are shown at the right margin. (B) Polar lipids from the indicated strains were separated by TLC and stained with the aminogroup-specific dye ninhydrin. Positions of PE and Lys-PG are indicated. (3.21 MB TIF) [file ppat.1000660.s004.tif]
