# Supplementary material for: The Bacterial Defensin Resistance Protein MprF Consists of Separable Domains for Lipid Lysinylation and Antimicrobial Peptide Repulsion
Source: PLoS Pathog. 2009 Nov 13;5(11):e1000660. doi: 10.1371/journal.ppat.1000660 (PMC2774229; doi:10.1371/journal.ppat.1000660)
Supplement: Table S1 — Plasmids for expression of truncated or mutated MprF variants. (0.02 MB PDF) [file ppat.1000660.s005.pdf]

Table S1. Plasmids for expression of truncated or mutated MprF variants.

Plasmid	Truncation or mutation introduced into MprF	Restriction Sites
pET28mprF	N-terminal His-tag fused; no truncations or point mutations; encompasses aa 1-840 of MprF; cloned in pET28a vector [2]	*Bam*H1/*Xho*I
pET28mprF(-2)	As pET28mprF but two TMSs removed from the N-terminus; encompasses aa 84-840 of MprF	*Bam*H1/*Eco*RI
pET28mprF(-4)	As pET28mprF but four TMSs removed from the N-terminus; encompasses aa 157-840 of MprF	*Bam*H1/*Eco*RI
pET28mprF(-6)	As pET28mprF but six TMSs removed from the N-terminus; encompasses aa 219-840 of MprF	*Bam*H1/*Eco*RI
pET28mprF(-8)	As pET28mprF but eight TMSs removed from the N-terminus; encompasses aa 274-840 of MprF	*Bam*H1/*Eco*RI
pET28mprF(-10)	As pET28mprF but ten TMSs removed from the N-terminus; encompasses aa 363-840 of MprF	*Bam*H1/*Hin*dIII
pET28mprF(-12)	As pET28mprF but twelve TMSs removed from the N-terminus; encompasses aa 437-840 of MprF	*Bam*H1/*Hin*dIII
pET28mprF(-14)	As pET28mprF but all 14 TMSs removed from the N-terminus; encompasses aa 510-840 of MprF	*Bam*H1/*Hin*dIII
pET28mprF(-8 D546A)	As pET28mprF(-8) but aspartate 546 replaced with alanine	*Bam*H1/*Eco*RI
pET28mprF(-8 K547A)	As pET28mprF(-8) but lysine 547 replaced with alanine	*Bam*H1/*Eco*RI
pET28mprF(-8 K621A)	As pET28mprF(-8) but lysine 621 replaced with alanine	*Bam*H1/*Eco*RI
pET28mprF(-8 E685A)	As pET28mprF(-8) but glutamate 685 replaced with alanine	*Bam*H1/*Eco*RI
pET28mprF(-8 E624A)	As pET28mprF(-8) but glutamate 624 replaced with alanine	*Bam*H1/*Eco*RI
pET28mprF(-8 D731A)	As pET28mprF(-8) but aspartate 731 replaced with alanine	*Bam*H1/*Eco*RI
pET28mprF(-8 R734A)	As pET28mprF(-8) but arginine 734 replaced with alanine	*Bam*H1/*Eco*RI
pET28mprF(-8 K806A)	As pET28mprF(-8) but lysine 806 replaced with alanine	*Bam*H1/*Eco*RI
pBADmprF	C-terminal His-tag fused; no truncations or point mutations; encompasses aa 1-840 of MprF; cloned in pBAD [3]	*Bgl*II/*Eco*RI
pBADmprF(D546A)	As pBADmprF but aspartate 546 replaced with alanine	*Bgl*II/*Eco*RI

pBADmprF(K547A)	As pBADmprF but lysine 547 replaced with alanine	*Bgl*II/*Eco*RI
pBADmprF(K621A)	As pBADmprF but lysine 621 replaced with alanine	*Bgl*II/*Eco*RI
pBADmprF(E685A)	As pBADmprF but glutamate 685 replaced with alanine	*Bgl*II/*Eco*RI
pBADmprF(E624A)	As pBADmprF but glutamate 624 replaced with alanine	*Bgl*II/*Eco*RI
pBADmprF(D731A)	As pBADmprF but aspartate 731 replaced with alanine	*Bgl*II/*Eco*RI
pBADmprF(R734A)	As pBADmprF but arginine 734 replaced with alanine	*Bgl*II/*Eco*RI
pBADmprF(K806A)	As pBADmprF but lysine 806 replaced with alanine	*Bgl*II/*Eco*RI
pRB474mprF(-8)	Truncated *mprF* gene encoding the MprF(-8) protein cloned in *staphylococcus*-specific expression vector pRB474 [7]; encompasses aa 274-840 of MprF	*Bgl*II/*Eco*RI
pTX15mprF(-8)	Truncated *mprF* gene encoding the MprF(-8) protein cloned in *staphylococcus*-specific expression vector pTX15 [8]; encompasses aa 274-840 of MprF	*Bgl*II /*Eco*RI
pTX15mprF(-C)	Truncated *mprF* gene encoding the MprF(-C) protein cloned in *staphylococcus*-specific expression vector pTX15 [8]; encompasses aa 1-586	*Bam*HI/*Eco*RI
