# Supplementary material for: The Bacterial Defensin Resistance Protein MprF Consists of Separable Domains for Lipid Lysinylation and Antimicrobial Peptide Repulsion
Source: PLoS Pathog. 2009 Nov 13;5(11):e1000660. doi: 10.1371/journal.ppat.1000660 (PMC2774229; doi:10.1371/journal.ppat.1000660)
Supplement: Table S2 — Primers used for plasmid construction. (0.02 MB PDF) [file ppat.1000660.s006.pdf]

Table S2: Primers used for plasmid construction.

Plasmid	Primers used for amplification or mutagenesis
pET28mprF	5´TAATTAGGATCCATGTGAAAAAATGAATCAGGAAGT<br>5´TACCTCGAGGCACTTGGATTTTAATTATTTG
pET28mprF(-2)	5´GATGGATCCTTAGGCAAAGTTTTAAGAG<br>5´ACCTCTTAGCACTTGGGAATTCATTATTTGTGACGTAT
pET28mprF(-4)	5´GTTTTCGATGGATCCTTAATCTTAGATAAG<br>5´ACCTCTTAGCACTTGGGAATTCATTATTTGTGACGTAT
pET28mprF(-6)	5´GGGATCCTTGACGCTCATGTATCATTCATG<br>5´ACCTCTTAGCACTTGGGAATTCATTATTTGTGACGTAT
pET28mprF(-8)	5´CCTGAGGAAAAAGTAGGATCCATGCTACTTCTATATCG<br>5´ACCTCTTAGCACTTGGGAATTCATTATTTGTGACGTAT
pET28mprF(-10)	5´CGATGCTGGATCCGACGGAAATCACTTAACG<br>5´CTAGAAAGCTAAGCTTTTATTTGTGACGTATTACACG
pET28mprF(-12)	5´TTATTGTAGGATCCCGTAGAGCACGTAGG<br>5´CTAGAAAGCTAAGCTTTTATTTGTGACGTATTACACG
pET28mprF(-14)	5´GGTATGATTGCAGGATCCTTTGATTATCAATTTAGC<br>5´CTAGAAAGCTAAGCTTTTATTTGTGACGTATTACACG
pET28mprF(-8)	5´CTTTAGGTGTCAGATCTGAGGAGGTATTATTAATGC<br>5´TACCTCGAGGCACTTGGATTTTAATTATTTG
pET28mprF(-C)	5´GATTTATAACAGAAAGGATCCGAGGAGGTGTGAAAAAATGAATCAGGA<br>5´GCCTTTGATGAATTGTTATAAGAATTCTATAATTACGC
pTX15mprF(-C)	5´GATTTATAACAGAAAGGATCCGAGGAGGTGTGAAAAAATGAATCAGGA<br>5´GCCTTTGATGAATTGTTATAAGAATTCTATAATTACGC
pTX15mprF(-8)	5’CTTTAGGTGTCAGATCTGAGGAGGTATTATTAATGC<br>5’CAAATAATTAGAATTCAACTGCTAAGAGGTA
pRB474mprF(-8)	5’CTTTAGGTGTCAGATCTGAGGAGGTATTATTAATGC<br>5’CAAATAATTAGAATTCAACTGCTAAGAGGTA
pBADmprF(D546A)	5'GATATATAGTGGTGCCAAGCAGTTTTTCACTAATGA<br>5'GCTGTTTTATTTTCATTAGTGAAAAACTGCTTGGCA
pBADmprF(K547A)	5'GATATATAGTGGTGACGCGCAGTTTTTCACTAATGA<br>5'GCTGTTTTATTTTCATTAGTGAAAAACTGCGCGTCA
pBADmprF(K621A)	5'CGGTAATCAATTTTTCGCATTAGGTGAAGAAGCAATTATTGATTTAACGC<br>5'GCTTCTTCACCTAATGCGAAAAATTGATTACCGAAATTATGATATAAAGG
pBADmprF(E685A)	5'GGCTAGATAATCGTCAGGCAATGCATTTCTCTGTTGG<br>5'CCAACAGAGAAATGCATTGCCTGACGATTATCTAGCC
pBADmprF(E624A)	5'CGGTAATCAATTTTTCAAATTAGGTGCAGAAGCAATTATTGATTTAACGC<br>5'GCGTTAAATCAATAATTGCTTCTGCACCTAATTTGAAAAATTGATTACCG
pBADmprF(D731A)	5'CCAACATACTTTAATGATGCCATTTCAGTCGCTTTAATTAGATGGTTGCC<br>5'GGCAACCATCTAATTAAAGCGACTGAAATGGCATCATTAAAGTATGTTGG
pBADmprF(R734A)	5'GCCATTTCAGTCGATTTAATTGCATGGTTGCCAGAGTTAGATTTACC<br>5'GGTAAATCTAACTCTGGCAACCATGCAATTAAATCGACTGAAATGGC
pBADmprF(K806A)	5'CGTTTCCAAGGATTACGTCGTTATGCATCTAAATATAATCCGAATTGGG<br>5'CCCAATTCGGATTATATTTAGATGCATAACGACGTAATCCTTGGAAACG
pET28mprF (-8 D546A)	5´CCTGAGGAAAAAGTAGGATCCATGCTACTTCTATATCG<br>5´ACCTCTTAGCACTTGGGAATTCATTATTTGTGACGTAT

pET28mprF (-8 K547A)	5´CCTGAGGAAAAAGTAGGATCCATGCTACTTCTATATCG<br>5´ACCTCTTAGCACTTGGGAATTCATTATTTGTGACGTAT
pET28mprF (-8 K621A)	5´CCTGAGGAAAAAGTAGGATCCATGCTACTTCTATATCG<br>5´ACCTCTTAGCACTTGGGAATTCATTATTTGTGACGTAT
pET28mprF (-8 E685A)	5´CCTGAGGAAAAAGTAGGATCCATGCTACTTCTATATCG<br>5´ACCTCTTAGCACTTGGGAATTCATTATTTGTGACGTAT
pET28mprF (-8 E624A)	5´CCTGAGGAAAAAGTAGGATCCATGCTACTTCTATATCG<br>5´ACCTCTTAGCACTTGGGAATTCATTATTTGTGACGTAT
pET28mprF (-8 D731A)	5´CCTGAGGAAAAAGTAGGATCCATGCTACTTCTATATCG<br>5´ACCTCTTAGCACTTGGGAATTCATTATTTGTGACGTAT
pET28mprF (-8 R734A)	5´CCTGAGGAAAAAGTAGGATCCATGCTACTTCTATATCG<br>5´ACCTCTTAGCACTTGGGAATTCATTATTTGTGACGTAT
pET28mprF (-8 K806A)	5´CCTGAGGAAAAAGTAGGATCCATGCTACTTCTATATCG<br>5´ACCTCTTAGCACTTGGGAATTCATTATTTGTGACGTAT
